# Penicillin causes non-allergic anaphylaxis by activating the contact system

**DOI:** 10.1038/s41598-020-71083-x

**Published:** 2020-08-25

**Authors:** Yuan Gao, Yixin Han, Xiaoyu Zhang, Qiaoling Fei, Ruijuan Qi, Rui Hou, Runlan Cai, Cheng Peng, Yun Qi

**Affiliations:** 1 Research Center for Pharmacology and Toxicology, Institute of Medicinal Plant Development, Chinese Academy of Medical Sciences and Peking Union Medical College, 151 North Ma Lian Wa Road, Haidian District, Beijing, People’s Republic of China; 2 Chengdu University of Traditional Chinese Medicine, Chengdu, 610075 People’s Republic of China

**Keywords:** Cell death and immune response, Coagulation system

## Abstract

Immediate hypersensitivity reaction (IHR) can be divided into allergic- and non-allergic-mediated, while “anaphylaxis” is reserved for severe IHR. Clinically, true penicillin allergy is rare and most reported penicillin allergy is “spurious”. Penicillin-initiated anaphylaxis is possible to occur in skin test- and specific IgE-negative patients. The contact system is a plasma protease cascade initiated by activation of factor XII (FXII). Many agents with negative ion surface can activate FXII to drive contact system. Our data showed that penicillin significantly induced hypothermia in propranolol- or pertussis toxin-pretreated mice. It also caused a rapid and reversible drop in rat blood pressure, which did not overlap with IgE-mediated hypotension. These effects could be countered by a bradykinin-B2 receptor antagonist icatibant, and consistently, penicillin indeed increased rat plasma bradykinin. Moreover, penicillin not only directly activated contact system FXII-dependently, but also promoted bradykinin release in plasma incubated-human umbilical vein endothelial cells. In fact, besides penicillin, other beta-lactams also activated the contact system in vitro. Since the autoactivation of FXII can be affected by multiple-factors, plasma from different healthy individuals showed vastly different amidolytic activity in response to penicillin, suggesting the necessity of determining the potency of penicillin to induce individual plasma FXII activation. These results clarify that penicillin-initiated non-allergic anaphylaxis is attributed to contact system activation, which might bring more effective diagnosis options for predicting penicillin-induced fatal risk and avoiding costly and inappropriate treatment clinically.

## Introduction

Anaphylaxis is a serious, life-threatening, generalized or systemic, allergy or non-allergic hypersensitivity with hypotension and vascular hyperpermeability as underlying symptoms^1,2^. Drugs are the most common anaphylaxis triggers in adults^3–5^. Beta­lactams, including penicillins and cephalosporins, are the most widely used antibiotics owing to their high safety profile, broad spectrum of activity, and low costs^6^. However, together with cephalosporins, penicillins are the antibiotics that most frequently provoke hypersensitivity reactions ranging from mild cutaneous symptoms to severe, life-threatening reactions^7^. Previous reports demonstrated that 42.6% of severe drug-induced anaphylaxis was caused by beta-lactam drugs^8^, and penicillin was the most frequent cause and accounted for approximately 75% of fatal anaphylactic cases in the United States^9^.

Penicillin, discovered by Alexander Fleming in 1928, is still one of the most widely prescribed antibiotics today. Clinically, approximately 10% of patients report a penicillin allergy (PenA), and its incidence of anaphylaxis has been estimated to be between 0.015 and 0.004%^10,11^. However, the reported PenA does not equate with true IgE-mediated allergy. In fact, true PenA is somewhat rare since only 1% of the general population is actually allergic to penicillin^12^. Most individuals thought to be PenA do not have detectable specific IgE (sIgE), and lack positive results from skin testing and oral challenge^13–16^. Only less than 10% of those with PenA histories are found to be at risk for acute PenA^17^. Some researchers ascribed the false attribution of PenA to IgE decline (disappearance) or viral rash misdiagnosis, while up to 90% of patients reporting PenA were found not to have a true allergy^10,12^. Obviously, it is unconvincing that such a high false rate is completely attributed to the above reasons. These data suggest that non-allergic mechanism(s) may be more common in the clinic.

The contact system is a plasma protease cascade initiated by activation of factor XII (FXII). Activated FXII (FXIIa) initiates two branches of downstream events that mediate the interface between coagulation and inflammation. The coagulation branch-activated FXI initiates fibrin formation and the inflammatory branch (also called kallikrein-kinin system)-activated prekallikrein cleaves the nonenzymatic cofactor high molecular weight kininogen (HK) to liberate oligopeptide bradykinin (BK)^18^. BK is involved in the regulation of inflammatory processes, vascular permeability, and blood pressure. Recent data have linked FXIIa-driven formation of BK and the downstream activation of the G-protein-coupled receptor B2 (B2R) signaling to anaphylaxis^19,20^. The name “contact system” comes from the mode of FXII being activated, as “contact” with negatively charged surfaces triggers FXII activation via conformational rearrangement. Activators that initiate FXIIa formation in vivo include polyphosphate, heparin, misfolded proteins, collagen, nucleic acids (DNA and RNA), oversulfated chondroitin sulfate and artificial surfaces^21,22^.

In the present study, we demonstrate for the first time that penicillin-driven FXII contact activation triggers kallikrein-kinin system, thus releasing BK to cause anaphylaxis. Our findings suggest that targeting contact system-produced BK or its downstream signaling is a promising strategy for prevention and treatment of anaphylaxis triggered by penicillin.

## Materials and methods

### Materials and reagents

Penicillin G sodium salt (1,550 U/mg) and other beta-lactams were from Solarbio Life Sciences (Beijing, China) and Yuanye Biotechnology Ltd. (Shanghai, China), respectively. Icatibant was from MedChemExpress (Monmouth Junction, NJ, USA). S-2302™ was from Chromogenix (Milano, Italy). BK ELISA kit was from DS Pharma Biomedical Co., Ltd. (Osaka, Japan). Kaolin, dextran sulfate sodium salt (DS; Mr ~ 500,000), propranolol, triprolidine, SB290157 and CV3988 were from Sigma-Aldrich (Darmstadt, German). PMX53 and pertussis toxin (PTX) were from GL Biochem Ltd. (Shanghai, China) and Enzo Life Sciences (Farmingdale, NY, USA), respectively. Citrate-anticoagulant standard human plasma and FXII-depleted plasma were from Boatman Biotech Co., Ltd. (Shanghai, China). Antibodies for human FXII and transferrin were from GeneTex Inc. (San Antonio, TX, USA). Shrimp tropomyosin (ST) and its IgE monoclonal antibody (mAb) were prepared as we previously described^23^. Human plasma was obtained from eight healthy volunteers.

### Cells and animals

Primary human umbilical vein endothelial cells (HUVEC) were from Lonza (Basel, Switzerland). The male Balb/c mice (18–20 g) and SD rats (180–200 g) were purchased from Vital River Experimental Animal Services (Beijing, China) and housed in an SPF laboratory.

### Anaphylactic shock assay

Anaphylactic shock was assessed by rectal thermometry^24^. To increase the severity of anaphylaxis, the mice were pretreated (i.v.) with propranolol (35 μg/mouse)^25,26^. Twenty minutes later, the mice were challenged (i.p.) with normal saline (NS) or penicillin (50 KU/mouse). Thirty minutes later, the rectal temperature was measured. For the BK antagonist experiment, icatibant (0.25 mM, 800 μL/mouse) was injected (i.p.) into the mice 10 min before the propranolol pretreatment.

The anaphylactic shock assay in PTX-pretreated mice was performed according to previously described procedures^27^. Briefly, the mice were injected (i.p.) with PTX (200 ng/mouse) on days 1 and 3. On day 7, the mice were injected (i.v.) with normal saline (NS) or penicillin (30 KU/mouse). Thirty minutes later, the rectal temperature was measured.

### Evans blue extravasation assays

Evans blue extravasation assays in mouse hindpaws and rat dorsal skin were measured as previously described^28,29^.

### Measurement of blood pressure and BK generation in rats

The rats were anesthetized and placed in a supine position. Catheters were placed into the carotid artery. Systolic arterial blood pressure was monitored in the left carotid artery with an arterial catheter connected to a polygraph (RM6240C, Chengdu, P.R. China). After equilibrating, the rats were injected (i.v.) with penicillin (1,250 KU/kg) or NS, and blood pressure was continuously monitored. For the antagonist experiment, rats were treated with icatibant (200 μg/kg, i.v.) 5 min prior to penicillin challenge.

For the determination of plasma BK, once penicillin (1,250 KU/kg) or NS was injected (i.v.) into the anesthetized rats, arterial blood was immediately collected and added to ice-cooled absolute ethanol. BK level was determined by ELISA.

The overlapping effect of penicillin- and antigen/IgE-caused hypotension was measured using a rat undergoing passive systemic anaphylaxis. To clarify the respective role of penicillin and antigen, we deliberately chose anti-ST IgE, a non-penicillin IgE mAb, to induce IgE/antigen hypotension. Rats were passively sensitized intravenously (i.v.) with 1 mg/kg of anti-ST IgE. Twenty-four hours later, the rats were challenged (i.v.) with ST (10 mg/kg) with or without penicillin (1,250 KU/kg), and the arterial blood pressure values were measured.

### Amidolytic activity assay

Amidolytic activity was assayed according to previously described procedures^30^. Briefly, 100 μL of plasma was pretreated with 100 μL of penicillin at various concentrations (diluted by Tris buffer: 50 mM Tris–HCl, 0.117 M NaCl, pH 7.8) at 37 °C. Ten minutes later, 100 μL of the chromogenic substrate S-2302 (1.5 mg/mL) was added and further incubated at 37 °C for 30 min. The reaction mix was centrifuged at 3,000 × *g* for 5 min. Supernatant absorbance was monitored at 405 nm. Kaolin and DS were used as positive control activators of the contact system. Buffer alone was included as a negative control.

### Determination of BK release in plasma incubated-human umbilical vein endothelial cells (PI-HUVEC)

The HUVEC were incubated with 10% standard human plasma in the presence of 20 μM Zn^2+^ at 37 °C for 1 h. The plasma was removed and the cells were washed twice. The obtained cells (PI-HUVEC) and non-PI-HUVEC were further incubated with penicillin at 37 °C for 30 min. Supernatant BK was determined by ELISA^18,31^.

### Data presentation

The data reported as the mean ± SD from a representative experiment. All of the experiments reported in this work were repeated at least three times with the same pattern of results. The statistical analysis was performed using a one-way ANOVA. A student’s *t*-test was used when only two groups were compared. *P* < 0.05 was considered significant.

### Ethic statement

Human plasma from healthy volunteers was obtained with written informed consent and all human studies were approved by the Ethic Review Board, Institute of Medicinal Plant Development of Chinese Academy of Medical Sciences. All the animal experiments were carried out according to the National Institutes of Health Guide for the Care and Use of Laboratory Animals and approved by the Institutional Care and Use Committee, Institute of Medicinal Plant Development of Chinese Academy of Medical Sciences. The informed consent form with official seal (the ethics approval number: 20180542x) for human blood collected from eight healthy volunteers. The animal ethics approval numbers are 20170728, 20171008, 20171234, 20180311 and 20180801. We confirm that all methods were performed in accordance with the relevant guidelines and regulations of our Ethic Review Board. Anesthetic drugs and all other necessary measures were used to reduce animal suffering during experimental procedures.

## Results

### Penicillin lowers rectal temperature in propranolol- or PTX-pretreated mice

**Figure 1:**
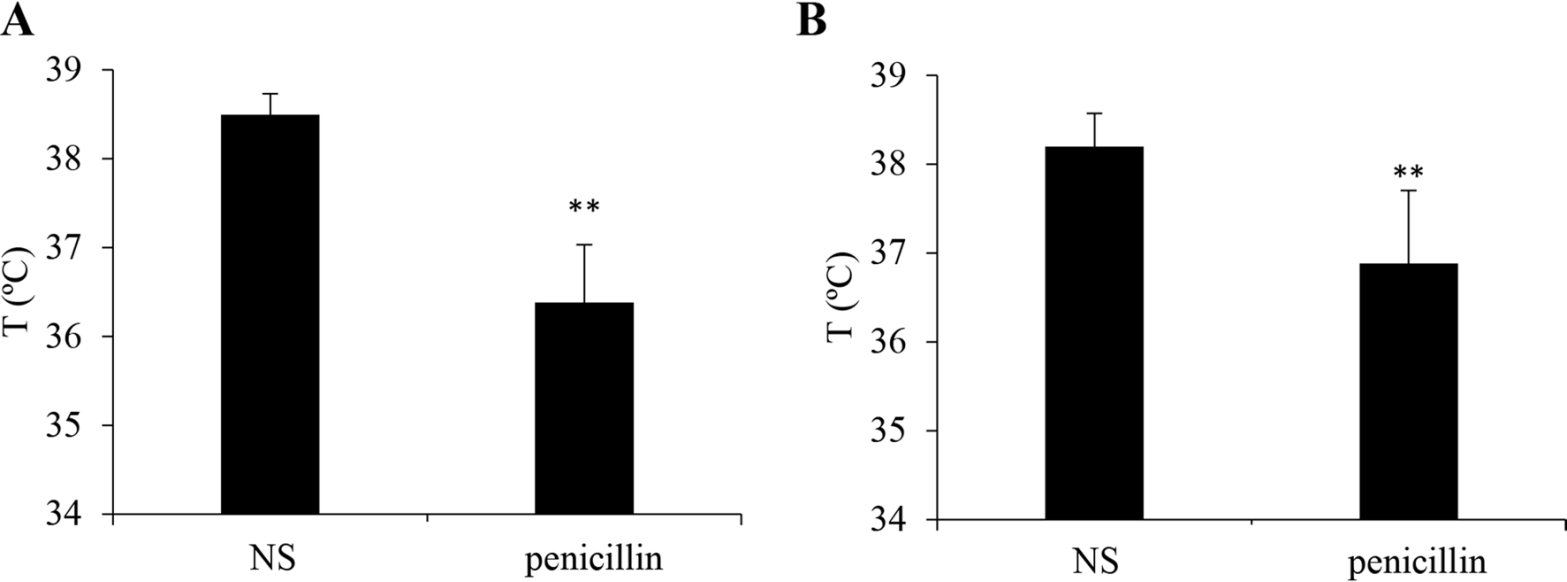
Penicillin induces anaphylactic shock in propranolol- or PTX-pretreated mice (n = 8). (**A**) Penicillin-induced hypothermia in propranolol-pretreated mice. The mice were pretreated (i.v.) with propranolol (35 μg/mouse). Twenty minutes later, the mice were injected (i.p.) with normal saline (NS) or penicillin (50 KU/mouse). Thirty minutes later, the rectal temperature was measured. **P < 0.01 vs. NS. (**B**) Penicillin caused hypothermia in PTX-pretreated mice. The mice were injected (i.p.) with PTX (200 ng/mouse) on days 1 and 3. On day 7, the mice were injected (i.v.) with NS or penicillin (30 KU/mouse). Thirty minutes later, the rectal temperature was measured. **P < 0.01 vs. NS.

Given that penicillin can provoke severe and life-threatening anaphylaxis clinically, we first evaluated whether penicillin could cause anaphylactic shock (detected as hypothermia)^7,24^. Mice were made more sensitive to penicillin-induced shock by pretreating propranolol or PTX, which does not induce shock by itself but can augment the susceptibility of shock via enhancing vascular permeability^25–27,32^. Penicillin contributed to obvious hypothermia in both propranolol- and PTX-pretreated mice (Fig. 1), indicating that penicillin can also cause a non-allergic anaphylactic shock.

### Penicillin increases microvascular permeability in a BK-dependent manner

**Figure 2:**
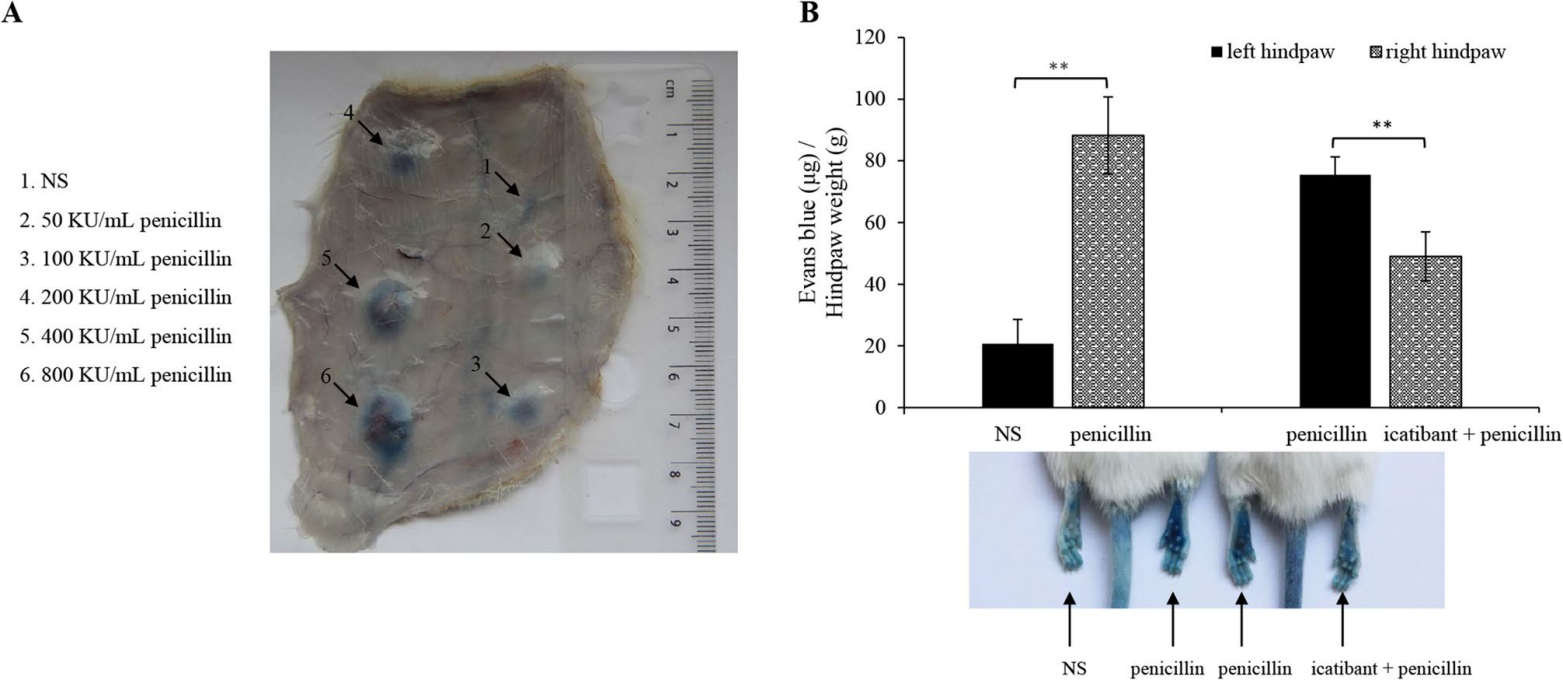
Penicillin increases microvascular permeability in a BK-dependent manner. (**A**) Representative image of Evans blue extravasation of rat dorsal inboard skin induced by penicillin (n = 3). Rats were anesthetized and intracutaneously injected with 100 μL of penicillin at different dosages and then immediately intravenously injected with 5 mg/mL of Evans blue (1 mL). Ten minutes later, the rats were euthanized and the resultant blue spots in the dorsal inboard skin were scored. A diameter > 5 mm was considered positive. (**B**) Icatibant blocked penicillin-induced local vasopermeability (n = 8). Fifteen minutes after induction of anesthesia (50 mg/kg of pentobarbital), mice were intraplantarly injected with 10 μL of icatibant (0.5 mM) or normal saline (NS). Twenty minutes later, the mice were injected (i.v.) with 50 μL of 12.5 mg/mL Evans blue. Five minutes later, 10 μL of penicillin (400 KU/mL) or NS was administered by intraplantar injection in the paw. Thirty minutes later, the mice were euthanized. The paw tissues were collected and weighed. Evans blue was extracted by a 24 h incubation in formamide at 60 °C. The OD values were read at 620 nm. The concentration of the dye in the paw tissues was calculated from the standard curve of the Evans blue dye, and the dye content was expressed in microgram per gram of tissue. **P < 0.01.

The local effect of penicillin on microvascular permeability was determined using the Evans blue extravasation assay. As shown in Fig. 2A, penicillin significantly (diameter of blue spot ≥ 5 mm) increased rat dorsal skin vasopermeability when the dose was ≥ 100 KU/mL (100 μL per spot). Moreover, it potently caused mouse plantar blue dye (right hindpaw) compared with NS (left hindpaw) (*P* < 0.01). To address the underlying mechanism, we next screened 6 antagonists (CV3988, SB290157, PMX53, triprolidine, fasudil and icatibant). The results showed that penicillin-increased microvascular permeability was independent of PAF, C3a, C5a, histamine and ROCK^33^ (data not shown), but could be countered by the B2R antagonist icatibant (Fig. 2B).

### Penicillin contributes to anaphylaxis by contact system activation (CSA) in rodents

**Figure 3:**
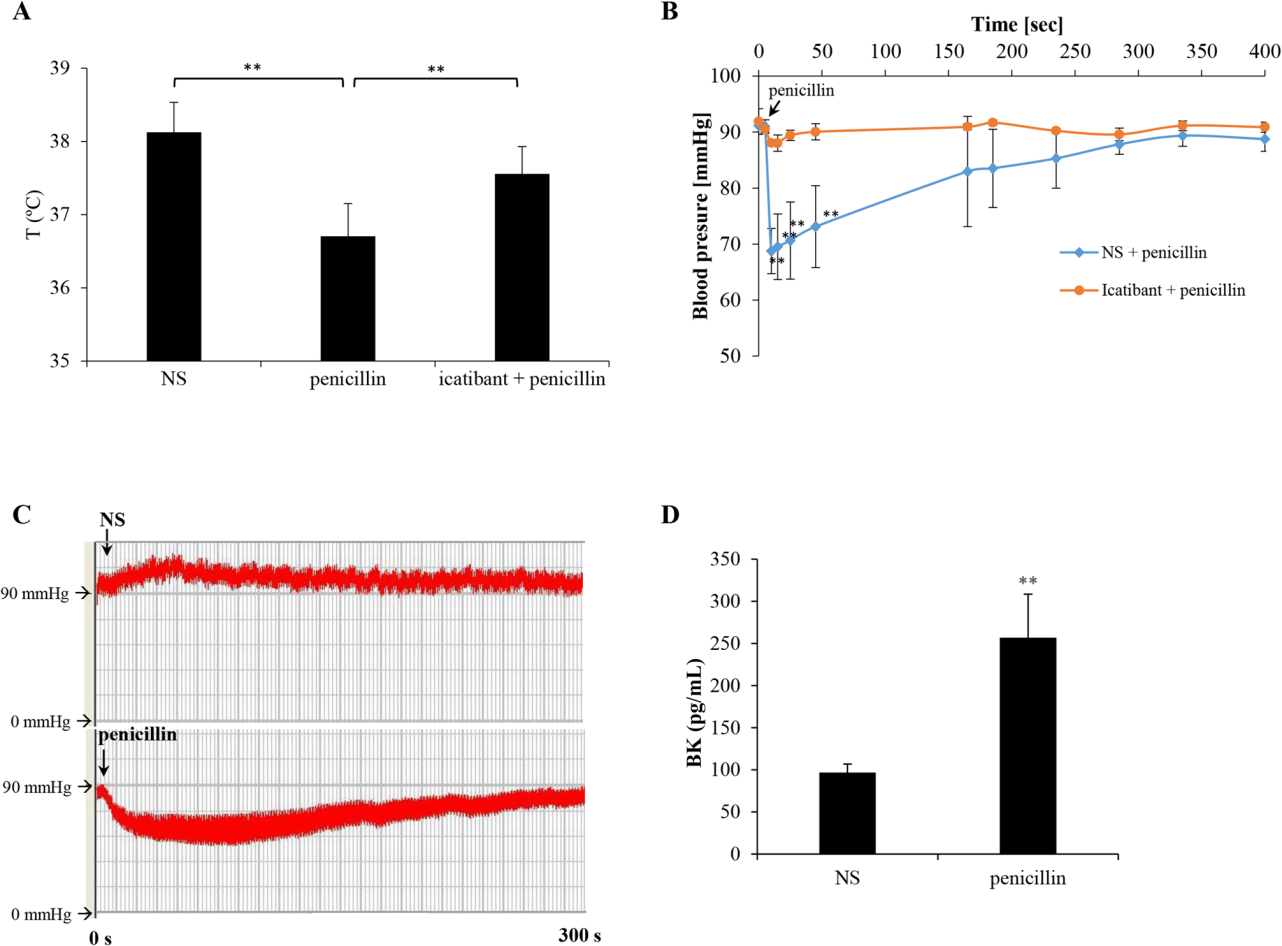
Penicillin contributes to anaphylaxis by activating the contact system in rodents. (**A**) Penicillin-induced hypothermia could be countered by icatibant in propranolol-pretreated mice (n = 8). **P < 0.01. (**B**) Penicillin induced a rapid and reversible drop in blood pressure in a BK-dependent manner (n = 6). The rats were pretreated with icatibant (200 μg/kg, i.v.) or normal saline (NS). Five minutes later, penicillin (1,250 KU/kg) was injected (i.v.) and the arterial blood pressure was measured in NS-pretreated (blue line) or icatibant-pretreated (orange line) rats. **P < 0.01 vs. icatibant + penicillin. (**C**) Typical tracing showing that penicillin induced a rapid and reversible drop in rat blood pressure. The rats were injected (i.v.) with NS or penicillin (1,250 KU/kg) and the arterial blood pressure was constantly monitored. (**D**) Penicillin significantly increased BK release in rat plasma. BK concentration was determined by a commercial ELISA kit. **P < 0.01 vs. NS.

Based on the above results, we next evaluated whether icatibant could block penicillin-caused non-allergic anaphylaxis. First, we evaluated the effect of icatibant on penicillin-induced hypothermia in propranolol-pretreated mice. As expected, icatibant indeed significantly countered penicillin-caused mouse hypothermia (*P* < 0.01; Fig. 3A). Given that acute hypotension is an underlying symptom of anaphylaxis^34^, the effect of penicillin on rat arterial blood pressure was then determined. Unlike intra-arterial administration of heparin^19^, intravenous injection of penicillin could trigger a rapid and transient drop in systemic arterial blood pressure. Pretreatment with icatibant almost completely blocked penicillin-caused hypotension (Fig. 3B,C). Along with the hypotension, plasma BK was markedly increased after penicillin challenge (Fig. 3D). These findings demonstrate that BK plays a crucial role in penicillin-initiated non-allergic anaphylaxis. In addition, to determine a possible overlapping effect of penicillin- and antigen/IgE-caused hypotension, SD rats were sensitized passively with a non-penicillin IgE (anti-ST IgE). The overlapping effect assay was carried out by intravenously injecting penicillin and antigen (ST) into the ST-IgE-sensitized rats. As a result (Table 1), penicillin and ST could respectively cause hypotension in normal and antigen/IgE-sensitized rats. Unexpectedly, penicillin did not cooperate with ST to trigger more drastic hypotension in antigen/IgE-sensitized rats.

**Table 1:** Mean arterial blood pressure values of normal or anti-shrimp tropomyosin (ST) IgE monoclonal antibody-sensitized rats in response to ST and/or penicillin (n = 5).

Challenge	Time (s)							
	0	10	30	60	120	180	240	300
Penicillin (1,250 KU/kg)^a^	91.2 ± 0.1	68.8 ± 4.0	70.1 ± 6.9	74.8 ± 7.3	82.9 ± 9.9	83.5 ± 7.0	85.3 ± 5.3	87.8 ± 1.8
ST (10 mg/kg)^b^	91.3 ± 0.8	71.3 ± 4.0	75.0 ± 3.6	78.8 ± 3.3	84.9 ± 4.4	85.6 ± 3.0	86.4 ± 3.5	88.7 ± 2.2
ST (10 mg/kg) + penicillin (1,250 KU/kg)^b^	90.6 ± 0.6	71.2 ± 4.4	71.7 ± 3.1	75.6 ± 2.1	81.2 ± 1.9	82.8 ± 3.1	86.5 ± 2.5	87.6 ± 2.3

^a^Challenge in normal rats.

^b^Challenge in anti-ST IgE monoclonal antibody-sensitized rats.

### Penicillin activates the contact system in an FXII-dependent manner

**Figure 4:**
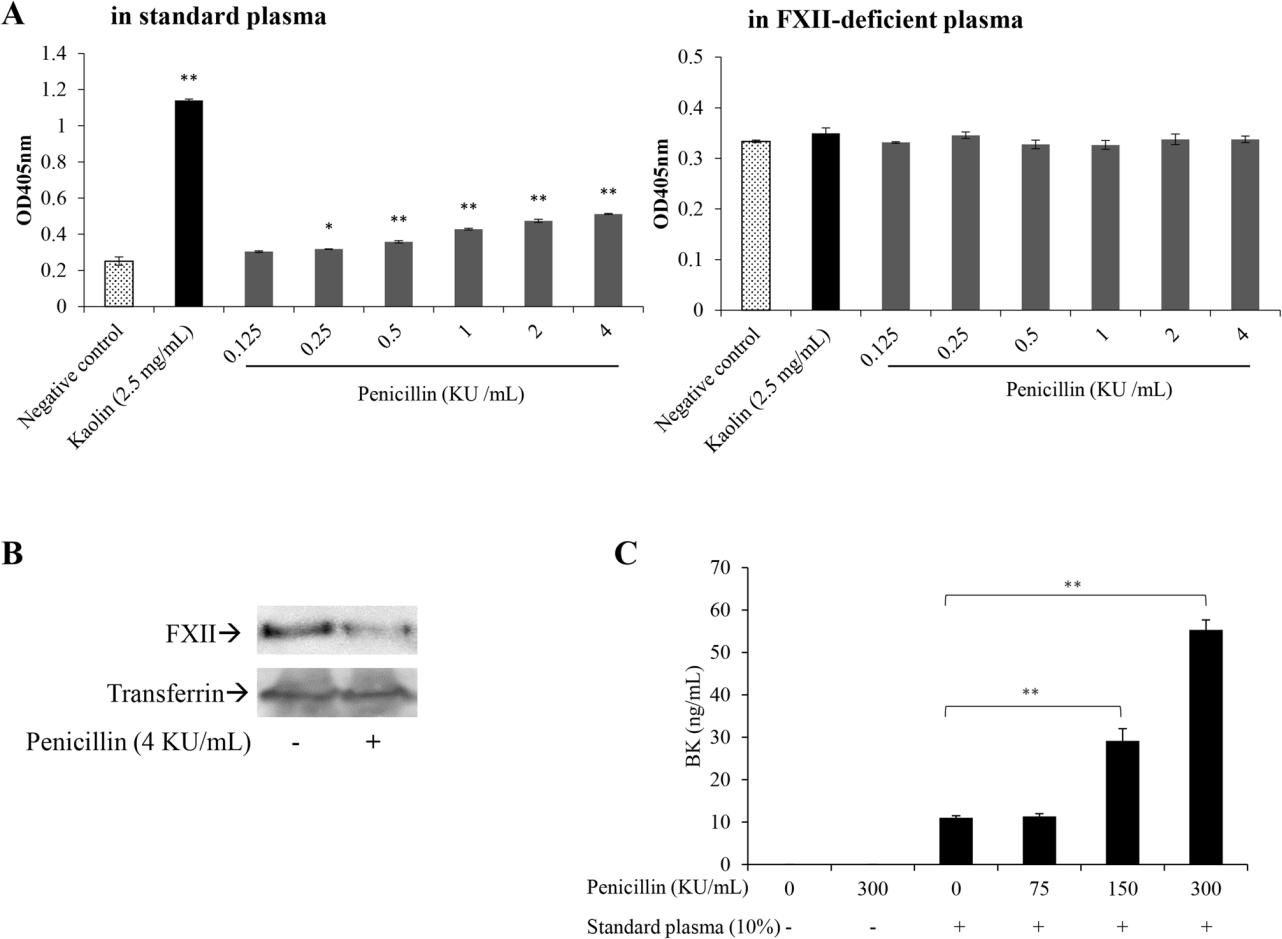
Penicillin activates the contact system in an FXII-dependent manner. (**A**) Penicillin-induced CSA in standard or FXII-deficient human plasma. 100 μL of plasma was pretreated with 100 μL of penicillin at various concentrations (diluted by Tris buffer: 50 mM Tris–HCl, 0.117 M NaCl, pH 7.8) at 37 °C. Ten minutes later, 100 μL of the chromogenic substrate S-2302 (1.5 mg/mL) was added and further incubated at 37 °C for 30 min. The reaction mix was centrifuged at 3,000 × *g* for 5 min. Supernatant absorbance was monitored at 405 nm. Kaolin was used as a positive control of the contact system. Buffer alone was included as the negative control. *P < 0.05 and **P < 0.01 vs. negative control. (**B**) Plasma prototypical FXII level decreased after penicillin treatment. Standard human plasma was incubated with or without 4 KU/mL penicillin at 37 °C for 30 min and analyzed for FXII determination by western blotting. Transferrin was used as the internal reference. Full-length blots and the detailed information of the used antibodies were presented in Supplementary file 1. (**C**) Penicillin induced BK release in PI-HUVEC. HUVEC were incubated with 10% standard human plasma in the presence of 20 μM Zn^2+^ at 37 °C for 1 h. The plasma was removed and the cells were washed twice. PI-HUVEC and non-PI-HUVEC were further incubated with penicillin at 37 °C for 30 min. Supernatant BK was determined by ELISA. **P < 0.01.

Direct activation of the contact system by penicillin was further investigated in vitro. Amidolytic activity was assessed in the standard plasma and FXII-deficient plasma by the addition of the S-2302 chromogenic substrate that can be hydrolyzed by plasma kallikrein. Similarly to the FXII activator kaolin, penicillin directly activated the contact system in standard human plasma, and this effect almost disappeared in FXII-deficient plasma (Fig. 4A). Consistently, owing to being converted into FXIIa consisting of a 52-kD heavy chain and a 28-kD serine protease domain, prototypical FXII (80-kD) in penicillin treated-plasma was also significantly decreased (Fig. 4B). BK, a short-lived bioactive peptide^35^, is the terminal product of CSA cascade. Given that the entire BK-forming cascade can be assembled and activated along the surface of endothelial cells^36,37^, HUVEC were incubated with standard human plasma and then the plasma was removed. The obtained PI-HUVEC, which had combined all elements for BK generation, could be used for the measurement of BK in vitro. As shown in Fig. 4C, unlike non-PI-HUVEC, PI-HUVEC could also release a small quantity of BK without penicillin. In contrast to non-PI-HUVEC, penicillin markedly promoted BK release in the PI-HUVEC in a concentration-dependent manner.

### Plasma from different individuals has distinct amidolytic activity

**Figure 5:**
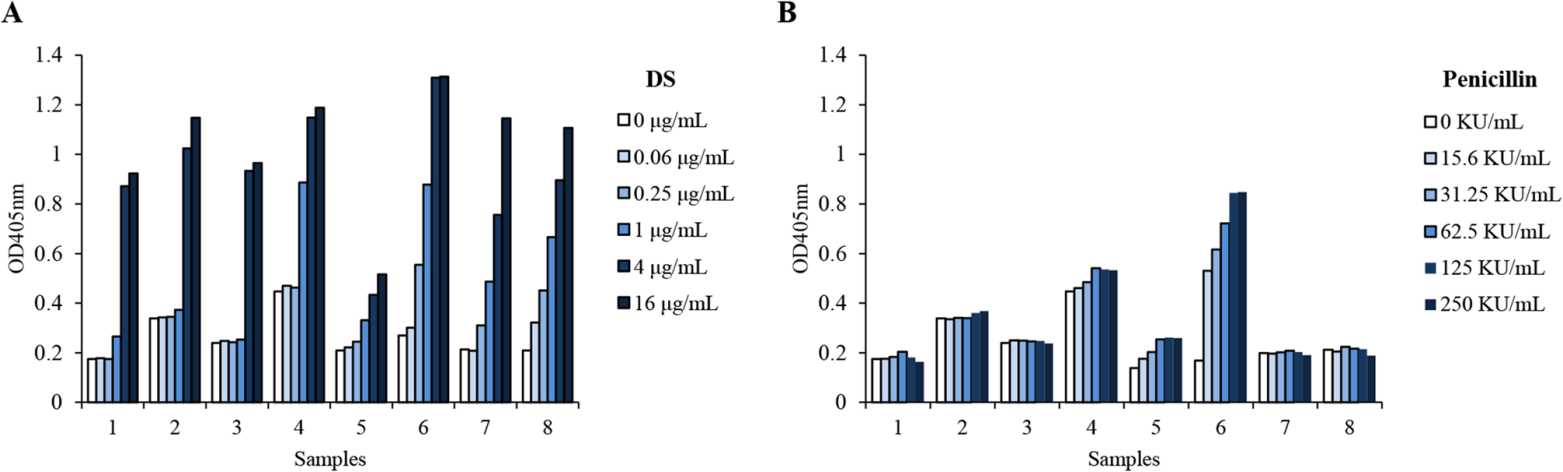
Plasma from different individuals has distinct amidolytic activity in response to DS (**A**) and penicillin (**B**). Human plasma was obtained from 8 healthy volunteers aged 20–54 years. 100 μL of plasma was pretreated with 100 μL of penicillin at various concentrations (diluted by Tris buffer: 50 mM Tris–HCl, 0.117 M NaCl, pH 7.8) at 37 °C. Ten minutes later, 100 μL of the chromogenic substrate S-2302 (1.5 mg/mL) was added and further incubated at 37 °C for 30 min. The reaction mix was centrifuged at 3,000 × *g* for 5 min. Supernatant absorbance was monitored at 405 nm.

The autoactivation of FXII-mediated CSA can be affected by various factors, including FXII^38^ and its endogenous inhibitor C1INH levels^39^, ionic environment (especially Zn^2+^ concentration^18,40^ or the negative charge density of FXII activators^41^), etc. Thus, we reasoned that plasma from different individuals had distinct amidolytic activity. To confirm this point, we assayed respective amidolytic activity in plasma from volunteers. As shown in Fig. 5, eight plasma samples (nos. 1–8) showed a consistent concentration–response to DS, including a relatively weak response of no. 5 (Fig. 5A). In contrast to DS, the majority responsiveness to penicillin was lower, except for no. 6. And nos. 1, 2, 3, 7 and 8 did not change significantly after penicillin treatment (≤ 250 KU/mL). Particularly, nos. 4 and 6, who had positive skin-test histories, reacted differently in response to penicillin: no. 4 had a high basal level but low sensitivity, while no. 6 had a low basal level but high sensitivity (Fig. 5B). These findings obviously indicate that the activation level of the contact system varies with diverse activators (e.g., penicillin and DS).

### Effects of other beta-lactams on CSA

**Figure 6:**
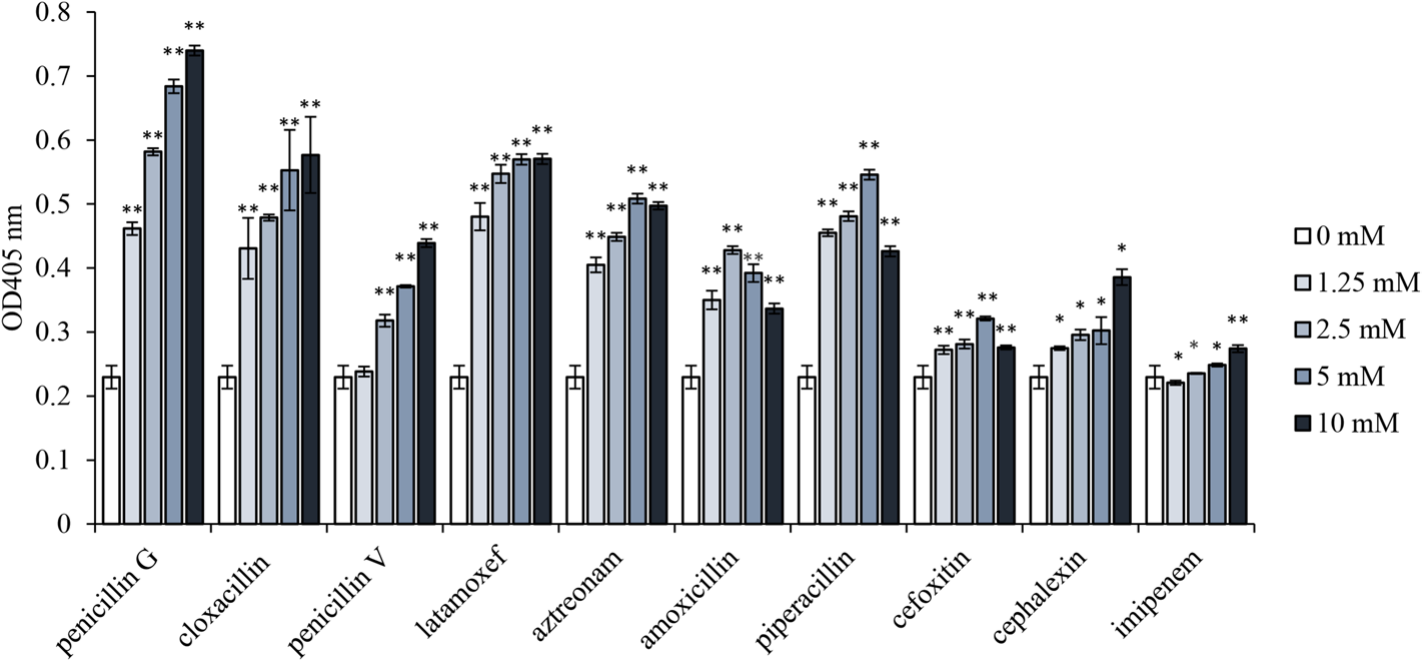
Effects of 10 beta-lactams on the amidolytic activity in standard human plasma. Beta-lactam antibiotics were incubated with standard human plasma at 37 °C for 10 min. Amidolytic activity in plasma was assayed using the chromogenic substrate S-2302. **P < 0.01 vs. negative control (0 mM).

To date, beta-lactams are the most successful chemical class of antibiotic widely used in the clinic to treat infections in humans^42^. They are mainly divided into 6 subclasses including penicillins (e.g., penicillin G, penicillin V, cloxacillin, amoxicillin and piperacillin), cephalosporins (e.g., cephalexin), oxacephems (e.g., latamoxef), single-ring beta-lactams (e.g., aztreonam), cephamycins (e.g., cefoxitin), and carbapenems (e.g., imipenem). We found that the effects of different beta-lactams on plasma amidolytic activity were various. Penicillin G was found to be the most effective, and cloxacillin, latamoxef and piperacillin had similar impacts. Other beta-lactams were found to be less effective (Fig. 6).

## Discussion

Immediate hypersensitivity reaction (IHR) can be divided into allergic- and non-allergic-mediated^43^. According to the World Allergy Organization proposition, “anaphylaxis” is reserved for severe IHR whose symptoms include hypotension, vascular leakage, or even cardiac arrhythmia and bronchial constriction in severe cases^20,44^. Previous studies have demonstrated that the FXII-driven contact system critically contributes to the pathogenesis of anaphylaxis in murine models^20^. Clinically, anaphylaxis is associated with transient decreased plasma FXII, suggesting that the factor is consumed. The severity of anaphylaxis is correlated with the intensity of CSA and BK formation^20^. The abnormal production of BK leads to anaphylaxis and angioedema via its ability to increase inflammation and vessel permeability^21,45^. There are common triggers for anaphylactic reactions such as food, medications or insect venom, of which 44–57% of the fatal anaphylaxis was triggered by medications, far more than other inducements^3,46–48^. The most common lethal reasons for fatal anaphylaxis are asphyxia and shock, which can be exactly caused by BK^49–52^.

**Figure 7:**
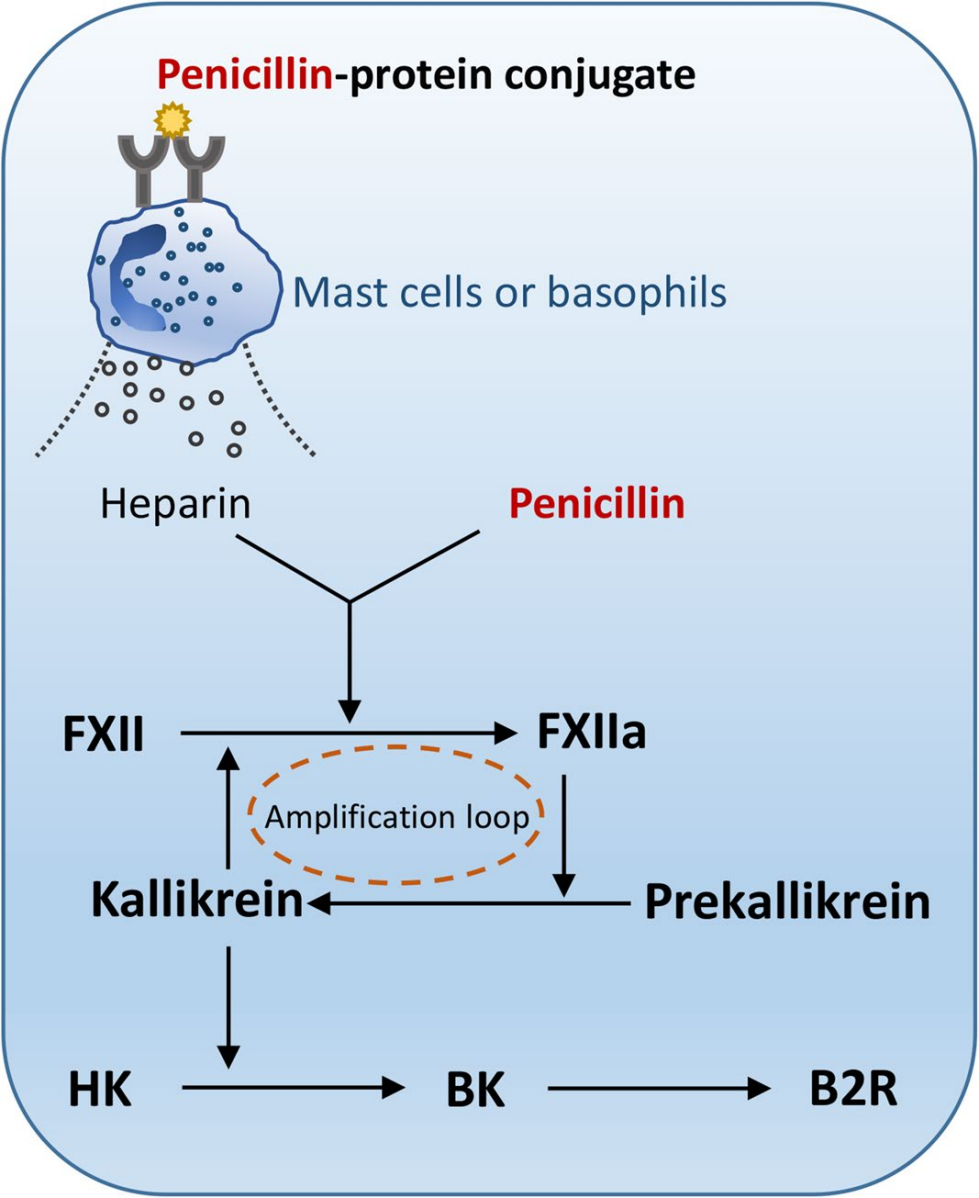
Penicillin triggers allergic- and non-allergic-mediated IHR via the FXIIa-BK-B2R axis.

An established concept is that penicillin can serve as a complete antigen to induce sIgE through irreversibly forming drug-protein conjugates in vivo^53,54^. Upon a secondary challenge, the penicillin-protein conjugate crosslinks its sIgE which bound to the high affinity receptor, FcɛRI, and leads to the degranulation of mast cells and basophils (Fig. 7), thus inducing type I hypersensitivity (e.g., anaphylaxis). In fact, true PenA is rare clinically, and most reported PenA is “spurious”^55,56^. Moreover, penicillin-induced IHR is possible to occur in both skin test- and sIgE-negative patients^57,58^, suggesting that a non-allergic mechanism exists. In fact, although penicillin could not directly induce human mast cell LAD2 degranulation in vitro (data not shown), it caused non-allergic anaphylaxis (Figs. 1, 3B). Our data showed that penicillin increased microvascular permeability, which could be countered by a B2R antagonist icatibant, rather than other antagonists (e.g., triprolidine and fasudil^33^) (Fig. 2B). Moreover, icatibant could also block penicillin induced-anaphylactic shock (Fig. 3A) and hypotension (Fig. 3B,C)*.* It is worth mentioning that heparin, an endogenous FXII activator, causes hypotension only through intra-arterial application^19^, which may be attributed to its rapid catabolism in blood circulation. In the present study, we found that penicillin triggered hypotension via both intra-arterial and intravenous injection. Given the fact that intra-arterial application of penicillin, an exogenous stimulus, is clinically meaningless, only the results of intravenous challenge were shown. In line with the in vivo result (Fig. 3D), in vitro study indicated that penicillin initiated CSA through the activation of FXII (Fig. 4A,B) and finally led to BK formation (Fig. 4C). Together, penicillin-initiated non-allergic anaphylaxis was attributed to CSA in which FXIIa activates prekallikrein leading to BK release via cleavage of its precursor HK.

The fact that penicillin can trigger both IgE- and non-allergic-mediated anaphylaxis raises the reasonable speculation about whether penicillin-induced severe or fatal anaphylaxis results from an overlapping effect. Unexpectedly, penicillin did not cooperate with antigen to trigger more drastic hypotension in antigen/IgE-sensitized rats (Table 1). In allergen/IgE-mediated type I hypersensitivity, mast cell or basophil-derived heparin can also initiate FXII contact activation and subsequently generate BK, which binds to B2R, triggering anaphylaxis^19,20^. Obviously, both allergen/IgE- and non-allergic-mediated anaphylaxis have the same pathway, namely the FXIIa-BK-B2R axis. Perhaps it is because the same axis that the overlapping effect did not occur. In addition, only one dose of penicillin was used in the rat blood pressure test due to the fact that penicillin caused hypotension in a non-dose-dependent manner (data not shown), which is similar to heparin^19^. As we know, FXIIa cleaves prekallikrein to form active kallikrein, which in turn reciprocally activates more FXII, forming a powerful activation amplification loop (Fig. 7). It is because of the “positive feedback loop” that, once initiated, the penicillin-induced cascade reaction reaches completion independent of the dosage of penicillin. In this context, other factors (e.g., plasma FXII or C1INH level, etc.) may be more crucial for reaction intensity.

FXII autoactivation can be influenced by multi-factors, such as FXII^38^ and C1INH levels^39^, ionic milieu^18,40^, or the negative charge density of FXII activators^41^, etc. Indeed, our data also showed that plasma from different human individuals had vastly different amidolytic activity in response to penicillin (Fig. 5B and Supplementary file 2), of which high responders might be the potential anaphylaxis sufferer (e.g., no. 6 in Fig. 5B and no. 5 in Supplementary file 2). In fact, some clinical evidences also support our findings. For example, penicillin-induced IHR was much more likely with parenteral administration than oral administration^58^. Angioedema, BK-mediated swelling^45^, is also common in penicillin-induced IHR^59,60^. Perhaps, not coincidentally, Caucasians, whose FXII levels are significantly higher than Japanese^61^, suffer from more penicillin-induced IHR compared with Asians including Japanese^60^.

Given that true PenA is rare clinically and the FXIIa-BK-B2R axis is the same pathway for penicillin-triggered allergic- and non-allergic-IHR (Fig. 7)^19^, compared with the commonly used skin test or sIgE assay, it is perhaps more effective to determine the potency of penicillin (or beta-lactams) to induce individual plasma FXII activation or BK formation for predicting the risk of penicillin-induced IHR. We notice that the World Health Organization has established a “first international standard for FXII, Plasma, human”^62^, which also ought to be beneficial for predicting the risk of penicillin-induced IHR, even including that of other beta-lactams (e.g., penicillin G, cloxacillin, latamoxef, piperacillin and aztreonam, etc.; Fig. 6). Additionally, in the same volunteer, the degree of FXII activation in response to penicillin at the same concentrations is disparate over time (no. 5 in Fig. 5 vs. Supplementary file 2), which might be attributed to the change of plasma milieus. Thus, instant prediction, rather than long-term predication, may be available for predicting the IHR risk of penicillin.

In summary, our study demonstrates, for the first time, that as an activator of the FXII-driven contact system, penicillin can lead to hypersensitivity reactions in rodent models, which can be hampered by icatibant, a B2R pharmacological inhibitor. These findings not only warrant further exploration of the penicillin-driven contact system as a source of therapeutic targets for treatment of anaphylaxis triggered by penicillin, but also might bring more effective diagnosis options for the prediction of penicillin-induced fatal risk.

## Supplementary information

Supplementary information.

## Data Availability

The datasets used and analyzed during the current study are available from the corresponding author on reasonable request.
