# Supplementary material for: Penicillin causes non-allergic anaphylaxis by activating the contact system
Source: Sci Rep. 2020 Aug 25;10:14160. doi: 10.1038/s41598-020-71083-x (PMC7447753; doi:10.1038/s41598-020-71083-x)
Supplement: Supplementary file 1 — Supplementary information. [file 41598_2020_71083_MOESM1_ESM.pdf]

Penicillin causes non-allergic anaphylaxis by activating the contact system

Yuan Gao[1], Yixin Han[1], Xiaoyu Zhang[1], Qiaoling Fei[1], Ruijuan Qi[1], Rui Hou[1], Runlan Cai[1], Cheng Peng[2,*] and Yun Qi[1,*]

[1]Institute of Medicinal Plant Development, Chinese Academy of Medical Sciences & Peking Union Medical College, Beijing, 100193, China.

[2]Chengdu University of Traditional Chinese Medicine, Chengdu, 610075, China.

*Corresponding authors.

Supplementary file 1- Full blot information for the figure 4B

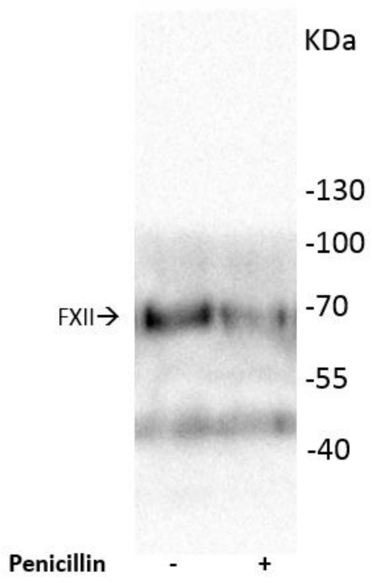

The information of FXII antibody:

Company: GeneTex;
Name: Factor XII antibody;
Catalog number: GTX53993; Lot No.821800170;
Package: 100 µg (1 mg/ml)
Website: https://www.genetex.com/Product/Detail/Factor-XII-antibody/GTX53993

Application Reference:

1. Lin CL et al. An occult hepatitis B-derived hepatoma cell line carrying persistent nuclear viral DNA and permissive for exogenous hepatitis B virus infection. PLoS One 2013; 8 (5):e65456.

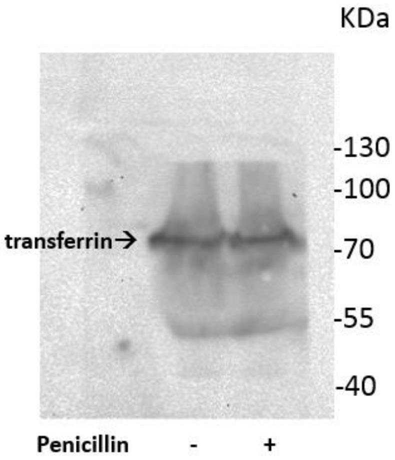

The information of Transferrin antibody:

Company: GeneTex;
Name: Transferrin antibody [N3C2], Internal;
Catalog number: GTX112729; Lot No.40093;
Package: 100 μl (0.92 mg/ml)
Website: https://www.genetex.com/Product/Detail/Transferrin-antibody-N3C2-Internal/GTX112729

Application Reference:

1. Marziali LN et al. Combined effects of transferrin and thyroid hormone during oligodendrogenesis In vitro. Glia 2016; 64 (11):1879-91.
2. Lin CL et al. An occult hepatitis B-derived hepatoma cell line carrying persistent nuclear viral DNA and permissive for exogenous hepatitis B virus infection. PLoS One 2013; 8 (5):e65456.

Supplementary file 2-Determination of the plasma from 5 healthy volunteers 1 year later.

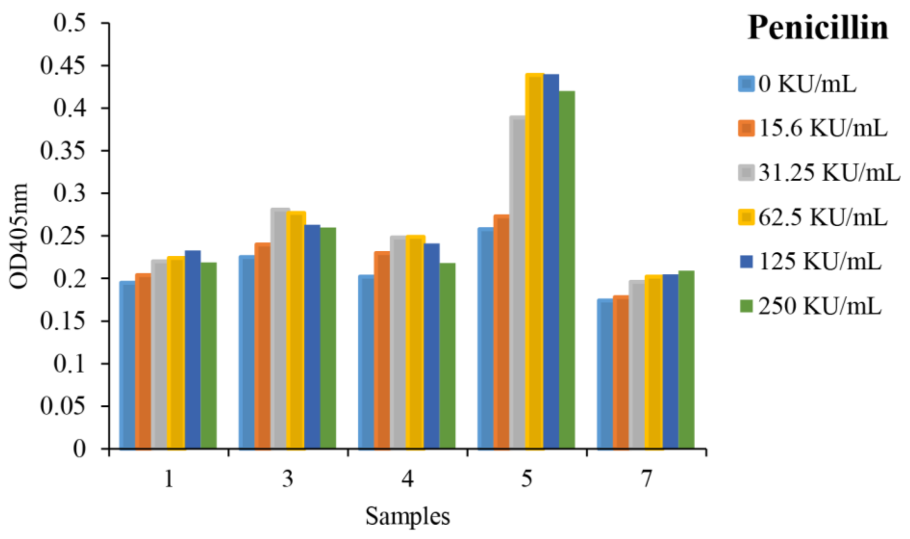

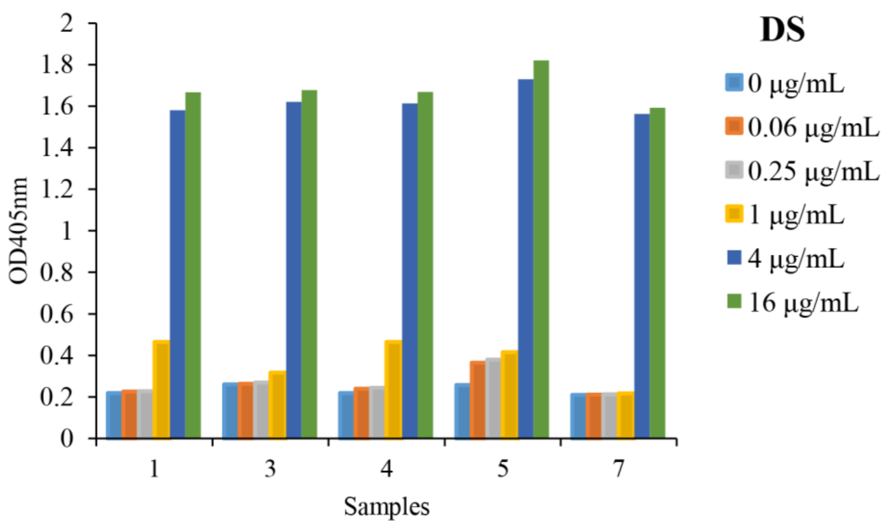

Plasma from different individuals has distinct amidolytic activity in response to DS (A) and penicillin (B). Human plasma was obtained from 5 healthy volunteers. 100 μL of plasma was pretreated with 100 μL of penicillin at various concentrations (diluted by Tris buffer: 50 mM Tris-HCl, 0.117 M NaCl, pH 7.8) at 37 ℃. Ten minutes later, 100 μL of the chromogenic substrate S-2302 (1.5 mg/mL) was added and further incubated at 37 ℃ for 30 min. The reaction mix was centrifuged at 3,000 × g for 5 min. Supernatant absorbance was monitored at 405 nm.
